# Down-Regulation of miR-92 in Breast Epithelial Cells and in Normal but Not Tumour Fibroblasts Contributes to Breast Carcinogenesis

**DOI:** 10.1371/journal.pone.0139698

**Published:** 2015-10-05

**Authors:** Laura Smith, Euan W. Baxter, Philip A. Chambers, Caroline A. Green, Andrew M. Hanby, Thomas A. Hughes, Claire E. Nash, Rebecca A. Millican-Slater, Lucy F. Stead, Eldo T. Verghese, Valerie Speirs

**Affiliations:** 1 Leeds Institute of Cancer and Pathology, University of Leeds, Leeds, United Kingdom; 2 Leeds Institute of Biomedical and Clinical Sciences, University of Leeds, Leeds, United Kingdom; 3 St James’s Institute of Oncology, St James’s University Hospital, Leeds, United Kingdom; King Faisal Specialist Hospital & Research center, SAUDI ARABIA

## Abstract

**Background:**

MicroRNA (miR) expression is commonly dysregulated in many cancers, including breast. MiR–92 is one of six miRs encoded by the miR-17-92 cluster, one of the best-characterised oncogenic miR clusters. We examined expression of miR–92 in the breast epithelium and stroma during breast cancer progression. We also investigated the role of miR–92 in fibroblasts in vitro and showed that down-regulation in normal fibroblasts enhances the invasion of breast cancer epithelial cells.

**Methodology/Principal Findings:**

We used laser microdissection (LMD) to isolate epithelial cells from matched normal, DCIS and invasive tissue from 9 breast cancer patients and analysed miR–92 expression by qRT-PCR. Expression of ERβ1, a direct miR–92 target, was concurrently analysed for each case by immunohistochemistry. LMD was also used to isolate matched normal (NFs) and cancer-associated fibroblasts (CAFs) from 14 further cases. Effects of miR–92 inhibition in fibroblasts on epithelial cell invasion in vitro was examined using a Matrigel™ assay. miR–92 levels decreased in microdissected epithelial cells during breast cancer progression with highest levels in normal breast epithelium, decreasing in DCIS (p<0.01) and being lowest in invasive breast tissue (p<0.01). This was accompanied by a shift in cell localisation of ERβ1 from nuclear expression in normal breast epithelium to increased cytoplasmic expression during progression to DCIS (p = 0.0078) and invasive breast cancer (p = 0.031). ERβ1 immunoreactivity was also seen in stromal fibroblasts in tissues. Where miR–92 expression was low in microdissected NFs this increased in matched CAFs; a trend also seen in cultured primary fibroblasts. Down-regulation of miR–92 levels in NFs but not CAFs enhanced invasion of both MCF–7 and MDA-MB–231 breast cancer epithelial cells.

**Conclusions:**

miR–92 is gradually lost in breast epithelial cells during cancer progression correlating with a shift in ERβ1 immunoreactivity from nuclei to the cytoplasm. Our data support a functional role in fibroblasts where modification of miR–92 expression can influence the invasive capacity of breast cancer epithelial cells. However in silico analysis suggests that ERβ1 may not be the most important miR–92 target in breast cancer.

## Introduction

MicroRNAs (miRs) are a class of short non-coding RNAs of 21–23 nucleotides that regulate gene expression and are commonly dysregulated in cancers, including those of the breast [1–3]. MiRs regulate expression of their target genes by binding to miR recognition elements, typically within 3’ untranslated regions (UTRs), causing translational inhibition and/or mRNA cleavage, thereby down-regulating expression of their protein products [4]. MiRs can also interact with coding regions and/or the 5’UTRs of their target transcripts suggesting numerous mechanisms by which these sequences can regulate gene expression [5, 6].

MiRs can function as oncogenes or tumour suppressors depending on their target genes. MiRs of the miR-17-92 cluster, also described as Oncomir–1, are thought to act as oncogenes and have been shown to promote cell proliferation and reduce apoptosis in lung cancer and lymphoma [7, 8]. There are 6 members of this cluster; miR–17, miR-18a, miR-19a, miR-20a, miR-19b-1 and miR-92a-1. Evidence suggests that these miRs exert their oncogenic role within cells by down-regulating the expression of specific anti-proliferative and/or pro-apoptotic genes including p63 [9], Bim [10] and components of the transforming growth factor (TGF)-β pathway [11]. In this regard, we have previously shown that expression of ERβ1 is negatively regulated by miR–92 in unselected non-microdissected breast cancers, providing a mechanism for down-regulation of this putative tumour suppressor gene [12]. More recently Nilsson et al. [13] found that high expression of miR–92 predicted better recurrence-free survival in breast cancer patients, an unexpected observation for a so-called onco-mir.

There is growing recognition that the tumour stroma can influence the behaviour of tumour cells, which may define patient outcomes [14, 15]. The most prominent change in breast stromal composition in response to tumourigenesis is an increase in the number of fibroblasts [16, 17]. These are the most common cell type in the breast tumour stroma and are usually known as cancer-associated fibroblasts (CAFs). A hallmark of CAFs is the much higher proportion of myofibroblasts within the total fibroblast population, identified by their expression of α-smooth muscle actin (α-SMA; [17, 18]). CAFs have been shown to increase tumour angiogenesis, tumour cell proliferation and the inflammatory response to the tumour. Examples of key molecules in CAF function include TGF-β [18], stromal cell-derived factor–1 (SDF–1; [17]) and phosphatase and tensin homolog (PTEN; [19]), which typically enhance tumourigenicity. Studies have uncovered numerous gene regulatory mechanisms responsible for dysregulation of such molecules in CAFs including changes in promoter methylation [20]and activity of key transcription factors [21]. However, the contributions of miRs to gene dysregulation in CAFs remain virtually unknown. In 2012, Zhao and colleagues reported the first miR expression profiling and identified key differences between CAFs and normal fibroblasts [22]. Likewise, Verghese et al. showed that miR-26b is down-regulated in CAFs from ER-positive breast cancers leading to enhanced epithelial cell migration and invasion [1]. The expression and function of miR–92 in breast stroma is yet to be addressed; however there is evidence to suggest that miR–92 expression is associated with macrophage infiltration in breast cancer indicating that this molecule might play an important role in tumour-stromal interactions [13].

Here, we aimed to examine expression of miR–92 in both the breast epithelium and stromal compartments during cancer progression and determine whether changing its expression in fibroblasts might modify the behaviour of breast cancer epithelial cells.

## Materials and Methods

### Ethics, Tissue and Laser Micro-Dissection (LMD)

Ethical approval was obtained from Leeds East research ethics committee (06/Q1206/180, project specific; 09/H1306/108, Leeds Breast Tissue Bank). Tissue samples were pseudo-anonymised and data were analysed anonymously. Fibroblasts were generated from fresh tissue obtained from patients who gave informed written consent (09/H1306/108). Formalin-fixed paraffin-embedded (FFPE) tissue blocks containing normal epithelium, DCIS and invasive breast lesions were obtained from the diagnostic archives of the Leeds Teaching Hospitals NHS Trust from 9 patients who had been treated for primary breast cancer in our centre. Under the terms of this project-specific ethics (06/Q1206/180), patient identities were not disclosed to the research team, hence specific informed consent was not required. Patient characteristics for these cases are shown in Table 1. H&E-stained sections were reviewed by a specialist breast pathologist (RM-S) for tissue verification. LMD was performed using a PALM microbeam microdissector using 100X or 200X magnification as described previously [1]. Areas of 5-10mm^2^ for each compartment were digitally outlined and catapulted into sterile opaque adhesive caps (PALM). Areas selected were devoid of visible cells other than target cell types. Normal (NF) and cancer-associated fibroblasts (CAFs) were isolated previously [1] from female patients with ER positive, HER–2 negative, grade 2 ductal NST cancers with no lymph node involvement.

**Table 1:** Pathological features of patients selected for LMD of normal, DCIS and invasive breast lesions (n = 9).

Case	Tumour type	Size (mm)	Age	Grade	ER	PR	HER2	LN
1	Ductal NST	26	69	2	+	-	-	N1
2	Ductal NST	14	55	2	+	+	-	N0
3	Ductal NST	20	45	2	+	+	-	N2
4	Ductal NST	16	69	1	+	+	-	N0
5	Ductal NST	14	40	2	+	+	-	N0
6	Ductal NST	55	61	3	-	-	-	N1
7	Ductal NST	27	86	2	+	+	-	N0
8	Ductal NST	28	43	2	+	+	-	N1
9	Ductal NST	30	48	1	+	+	-	N0

### Tissue Culture, Transfection, Transduction and Functional Assays

MCF–7, MDA-MB–231, T47D, HB2, BT–474, MDA-MB–453, MDA-MB–468 and BT–20 cells were maintained in RPMI 1640 medium, supplemented with 5% or 10% heat-inactivated fetal bovine serum (FBS; both Invitrogen), in a 5% CO_2_ humidified incubator at 37°C. Bimonthly *Mycoplasma* checks (MycoAlert Mycoplasma detection assay, Lonza) were consistently negative and short tandem repeat profiles confirmed cell identity (last tested April 2014). Primary fibroblasts were isolated from breast surgical samples and maintained in DMEM, supplemented with 10% heat-inactivated FBS, in a 5% CO_2_ humidified incubator at 37°C [23]. Reverse transfection of ERβ1 and non-targeting control siRNAs (Thermo Scientific), *mir*Vana™ miR–92 inhibitor and negative control #1 (Ambion; #MH10916 and #4464076) or miRNASelect™ pEP-hsa-mir-92a-1 expression vector (Cell Biolabs, Inc. #MIR-92A-1) and null control was performed using Lipofectamine 2000 or Lipofectamine RNAiMAX (both Invitrogen). Invasion assays were performed as described previously [1, 23]. Briefly, 5x10^4^ primary fibroblasts were reverse transfected (cells added directly to pre-prepared transfection agents) in 24 well plates and incubated at 37°C for 8h after which the transfection mix was removed and was replaced with fresh serum-free medium. Meanwhile, 1x10^4^ serum-starved MCF7 or MDA-MB–231 cells were seeded in serum-free medium into 24 well inserts coated with a Matrigel™ layer and incubated at 37°C for 2h. After incubation, these inserts were placed inside the wells which contained the transfected fibroblasts and co-cultured for 48h prior to fixation of culture inserts and subsequent visualisation with crystal violet (5mg/ml crystal violet, 50% methanol, 20% ethanol, 30% H_2_0), prior to analysis.

### RNA Extraction and Quantitative PCR Analysis

Total RNA extraction was achieved using the *mir*Vana™ miRNA Isolation Kit (Ambion). cDNA synthesis was performed using MegaPlex RT primers (Life Technologies) and a Megaplex pre-amplification step was undertaken for all analyses from FFPE tissues (Life Technologies). qRT-PCR analyses were performed on 7500/7900HT machines in triplicates with Taqman assays (Life Technologies). Relative miR–92 expression was determined using normalisers U6 and RNU48.

### DNA Extraction and Pyrosequencing

DNA extraction was achieved using the DNeasy Blood and Tissue Kit (Qiagen). Primers for amplification and pyrosequencing analysis of the methylation status of MIR17HG were designed using proprietary pyrosequencing Assay Design Software v2.0 (Qiagen). Primer sequences are in Table 2. These primers were used to analyse all 10 CpG’s between chr13:91348952 and chr13:91348988. Assay designs were saved as.*xml* files and imported to Pyro Q-CpG Software v1.0.9, which generated a nucleotide dispensation order according to the manufacturer’s standard parameters. Suitable bisulphite treatment controls were chosen from the options provided by the software. PCR reactions contained 12.5μl of Qiagen HotStarTaq Master Mix (Qiagen), additional magnesium chloride to achieve a final concentration of 2mM, 200nM each of forward and reverse primers, 2ul of DNA eluted from the bisulphite conversion and sufficient water to make a final volume of 25μl. Thermal cycling conditions were 94°C for 12 minutes to activate the Taq polymerase followed by 40 cycles of 94°C for 10 seconds, 55°C for 20 seconds and 72°C for 30 seconds. PCR products were sequenced by pyrosequencing on a PyroMark ID system (Qiagen) following the manufacturer’s protocols. Percentage methylation at each of the four CpG sites was calculated by the Pyro Q-CpG Software. Pyrograms and analysis reports were exported from this software.

**Table 2:** Primers used for pyrosequencing.

Primer	Sequence (5’-3’)	Genomic co-ordinates (GRCh38/hg38)
Forward	GGGGGTTGGGGGATATAAA	chr13:91348926–91348944
Reverse (biotinylated)	CCTTTTTCAATTCCTTTTCCCTTTAC	chr13:91349234–91349209
Sequencing	GGGGGATATAAAGGAG	chr13:91348933–91348948

### Immunohistochemistry

Immunohistochemistry was performed as previously described [24]. Briefly, sections were dewaxed with xylene and rehydrated through graded ethanol before blocking of endogenous peroxidase activity in 3% H_2_O_2_ (10min). Epitopes were retrieved by heating in a pressure-cooker in 1% vector antigen unmasking solution (2min) and non-specific binding blocked using 10% Casein solution (20min). Slides were incubated with mouse anti-ERβ1 antibody (clone PPG5/10, Serotec) at a 1:20 dilution for 16h at 4°C. Staining was visualised using Envision kits (Dako, Gostrup, Denmark). Slides were washed in tris-buffered saline and stained in copper sulphate, Harris’ haematoxylin and finally in Scotts substitute for 1min before dehydration. Slides were mounted in DPX (Fluka, UK). Stained sections were digitally scanned using Scanscope XT (Aperio) at 40x magnification and were observed using ImageScope (Aperio). Nuclear immunoreactivity was scored as percentage of positive cells in relation to total number of tumor cells present and using the Allred score based on nuclear staining intensity and proportion of positively stained nuclei, which generates numerical values from 0 to 8 [25]. Cytoplasmic staining was determined according to [24], where 0, no staining; 1, weak; 2, moderate; 3, strong. Cases were scored independently by two observers (LS and VS). Discordant results were reevaluated jointly to reach consensus.

### Data Mining and Statistical Analyses

BreastMark [26] and Oncomine [27]platforms were used for data mining. High and low expression of candidate miR–92 targets was quantified as above or below mean expression for that gene in all patients, respectively. Statistical analyses were performed using Prism (GraphPad) with tests (two-tailed) as described in the text. P values of less than 0.05 were considered significant.

## Results

### Reduced Expression of miR–92 in the Breast Cancer Epithelium Is Associated with Cancer Progression and Poor Prognosis

**Fig 1:**
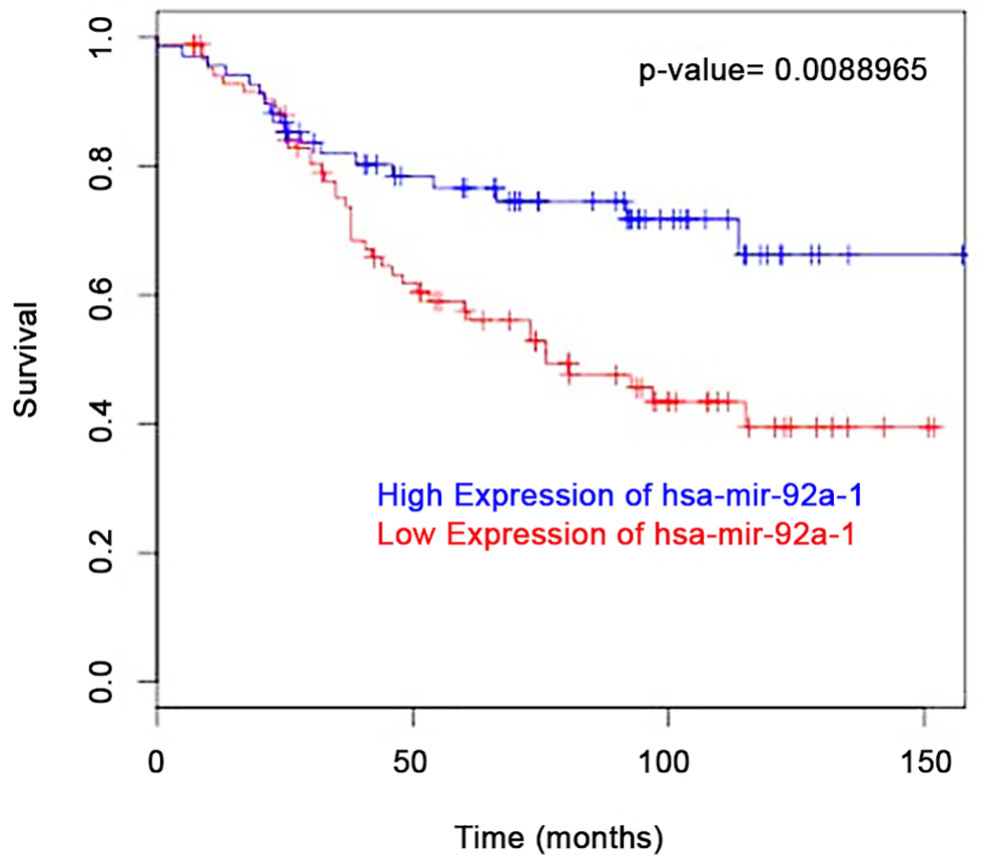
*In silico* analysis using BreastMark [26] shows luminal A breast cancer patients (n = 154) who express miR–92 have improved disease free survival (DFS) rate compared to patients with low expression levels with a hazard ratio of 0.49 (95% confidence intervals 0.28–0.84; p = 0.008).

As Nilsson et al. found that high expression of miR–92 was associated with better patient outcome [13] we performed an *in silico* analysis using the BreastMark platform [26]. Similarly, we found those patients with luminal A breast cancers who expressed high levels of miR–92 had a better disease free survival (DFS) rate compared to patients expressing low levels (Fig 1); an observation not expected for a so-called onco-mir.

**Fig 2:**
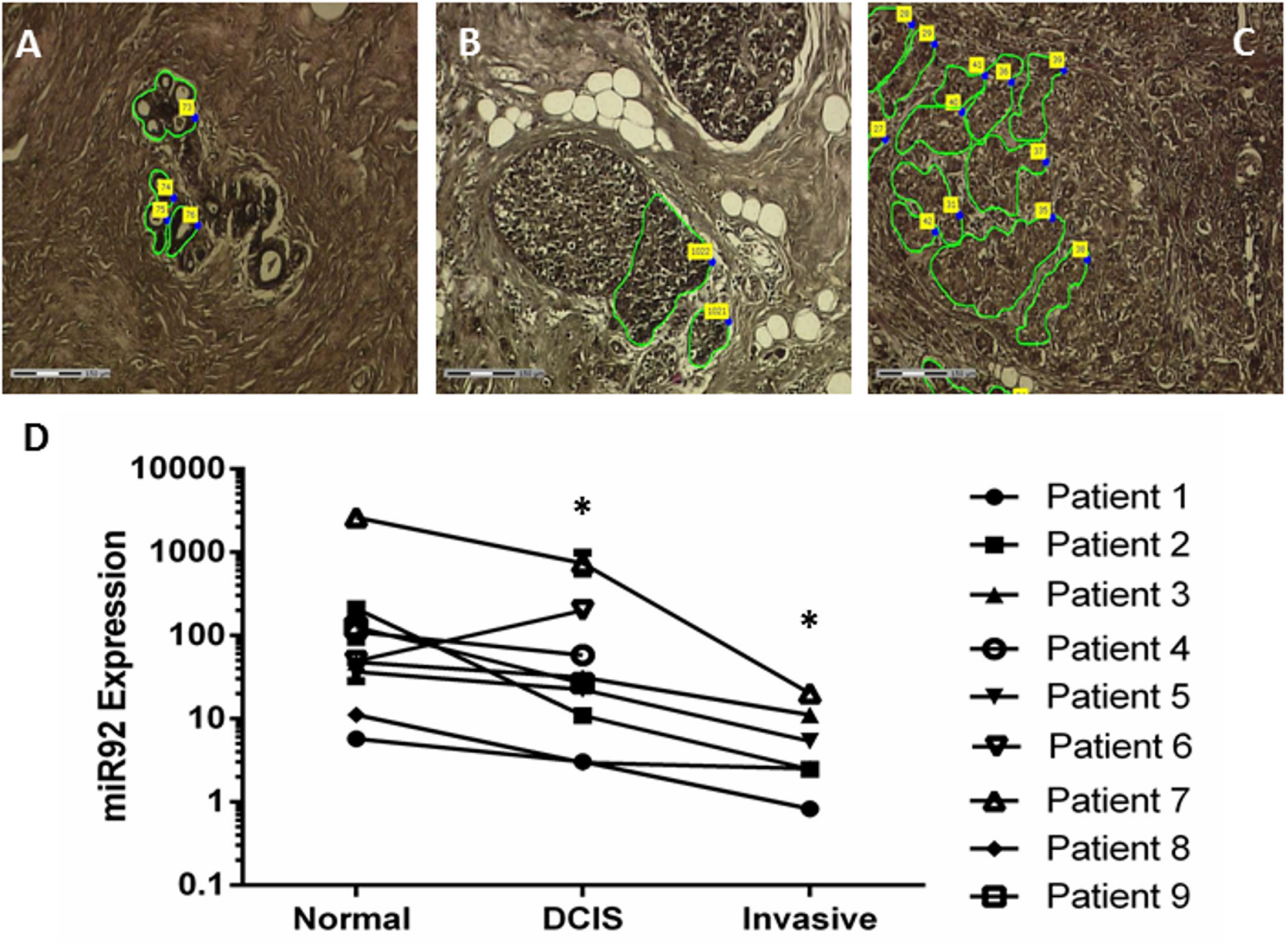
Laser micro-dissection (LMD) was used to isolate areas of epithelium in normal (A), DCIS (B) and invasive (C) breast tissue from the same tissue section. Breast tissue images show examples of the areas captured for patient 4 as a representative example. The expression of miR–92 decreased during breast cancer progression with highest levels observed in normal breast epithelium, decreasing in DCIS and being lowest in invasive breast tissue (D). *denotes significance of p<0.01.

We then examined expression levels of miR–92 in the breast epithelium during cancer progression. Our previous work had analysed miR–92 expression in unselected non-microdissected breast tissues. Here, we used LMD to isolate areas of matched normal, DCIS and invasive tissue from 9 patients with primary breast cancer (Fig 2A–2C) to specifically analyse expression in breast epithelial cells. Expression of miR–92 was determined by qRT-PCR after normalisation to U6, RNU48 or the geomean of both house-keepers. We were able to determine expression in all breast lesions for 6 patients. For 3 patients, we were unable to accurately determine expression levels from invasive breast tissue due to no detectable expression of either house-keeper for normalisation. For these 3 patients, we were able to calculate miR–92 expression in matched normal and DCIS breast tissues. We found that whilst absolute expression levels varied from patient to patient, the pattern was consistent for all but 1 case (patient 6). Overall, miR–92 levels decreased in epithelial cells during breast cancer progression with highest levels observed in normal breast epithelium, decreasing in DCIS (p<0.01) and being lowest in invasive breast tissue (p<0.01; Fig 2D).

### Expression of miR–92 Is Associated with an Altered Subcellular Location of ERβ1; a Direct miR–92 Target

**Fig 3:**
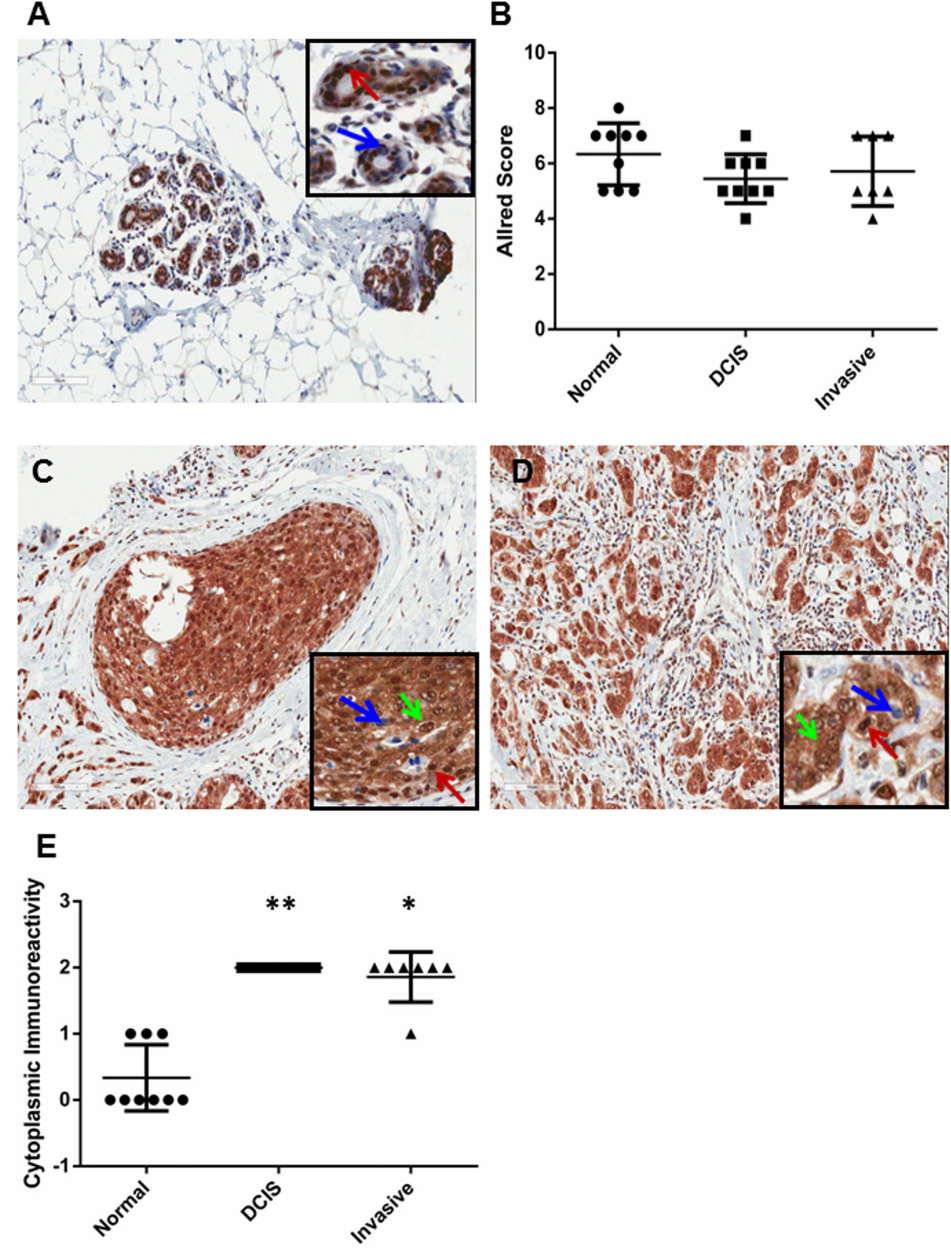
Expression of ERβ1 and miR–92 are not inversely correlated in patients with primary breast cancer. Staining patterns of ERβ1 showed nuclear expression in the normal breast (A) epithelium (examples shown by red arrows) with no significant difference in nuclear staining during cancer progression (B). A shift in the localisation of ERβ1 staining was observed with a significant increase in cytoplasmic staining (green arrows) during progression to DCIS (C, E) and invasive breast cancer (D, E). Blue arrows show ERβ1-negative nuclei. Images of breast tissues show the staining patterns for patient 4 as a representative example. Horizontal lines represent the mean. Range ± S.D. *denotes significance of p<0.05; **denotes significance of p<0.01.

Next, we sought to demonstrate whether the down-regulation of miR–92 was inversely related with ERβ1 expression in our patient cohort. We used an immunohistochemical approach to determine ERβ1 protein expression. This was required due to our previous findings that expression of ERβ1 is regulated by post-transcriptional mechanisms [28, 29], therefore we considered protein analysis would provide a more accurate correlation. Nuclear and cytoplasmic immunoreactivity was assessed. ERβ1 showed predominantly nuclear expression in the normal breast epithelium (Fig 3A) with no significant difference in nuclear staining through progression to DCIS and invasive breast cancer (Fig 3B). Interestingly we observed a shift in the localisation of ERβ1 immunoreactivity during breast cancer progression with a significant increase in cytoplasmic staining observed in DCIS (p = 0.0078; Fig 3C and 3E) and invasive breast lesions (p = 0.031; Fig 3D and 3E) compared with normal breast tissue. This suggests that miR–92 might influence the subcellular location of ERβ1. No changes were observed in the level of staining, only in sub-cellular location.

### Down-Regulation of miR–92 Expression in Normal Breast Fibroblasts Can Influence Behaviour of Breast Cancer Epithelial Cells

**Fig 4:**
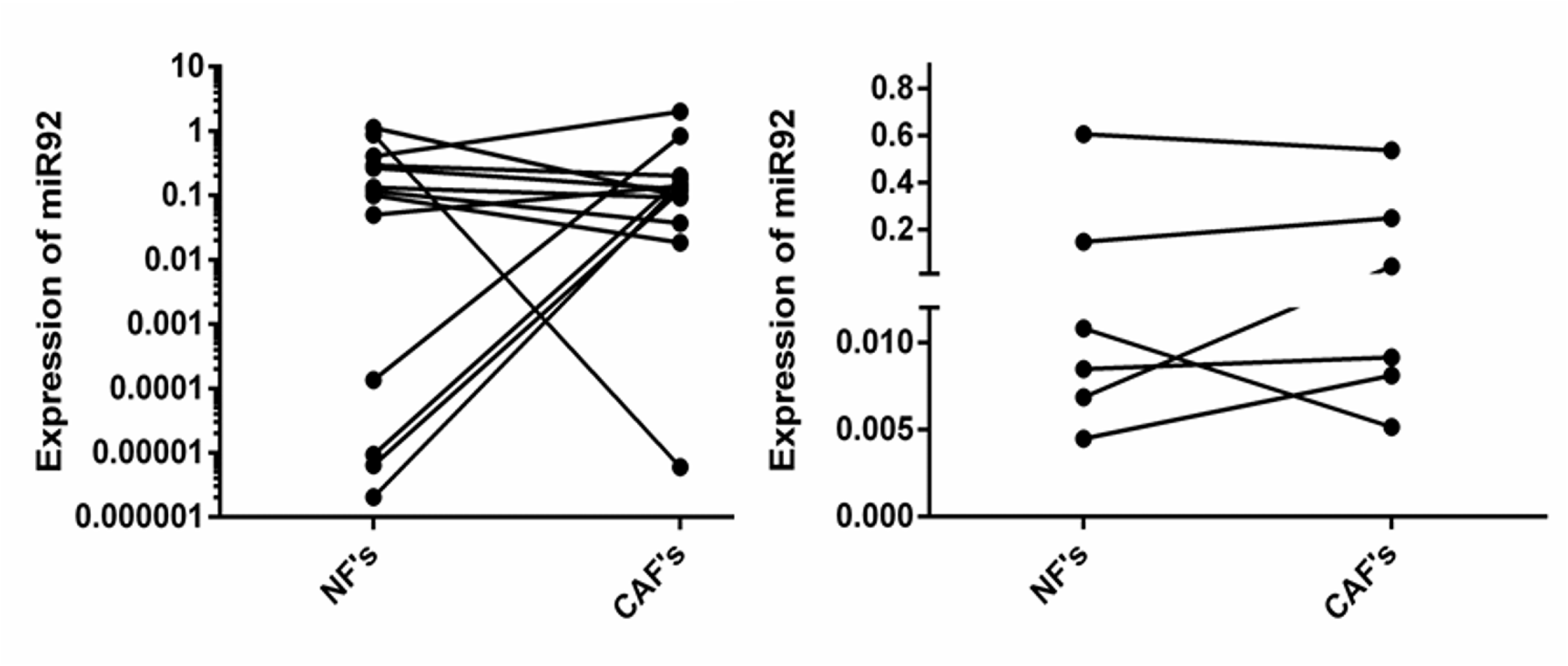
Expression of miR92 in normal fibroblasts (NFs) and cancer associated fibroblasts (CAFs) in patient samples (A) and primary fibroblast cultures (B) by qRT-PCR. *denotes significance of p = 0.0014 between NFs (high and low expressing groups) and NFs and CAFs (ANOVA).

As miR–92 levels decreased in the epithelial component during breast cancer progression we therefore examined the expression level of miR–92 in matched normal fibroblasts (NFs) and cancer-associated fibroblasts (CAFs) previously micro-dissected from 14 patients with ER-positive primary breast cancer [1]. CAFs were micro-dissected from the tumour block and NFs from a block of adjacent normal tissue from the same case. We also analysed expression in 6 cases of matched primary fibroblasts [1, 23]. miR–92 was stratified into 2 distinct groups of high and low miR–92 expression in NFs (Fig 4A). Interestingly, expression of miR–92 was markedly and significantly elevated in CAFs matched with NFs from the low expressing group. In contrast, miR–92 expression showed little variation in NFs and CAFs from the high-expressing group. This pattern was also observed in primary fibroblast cultures (Fig 4B). ERβ1 immunoreactivity was also seen in stromal fibroblasts, however the spindle-like shape of these cells made it impossible to distinguish nuclear and cytoplasmic staining (data not shown).

**Fig 5:**
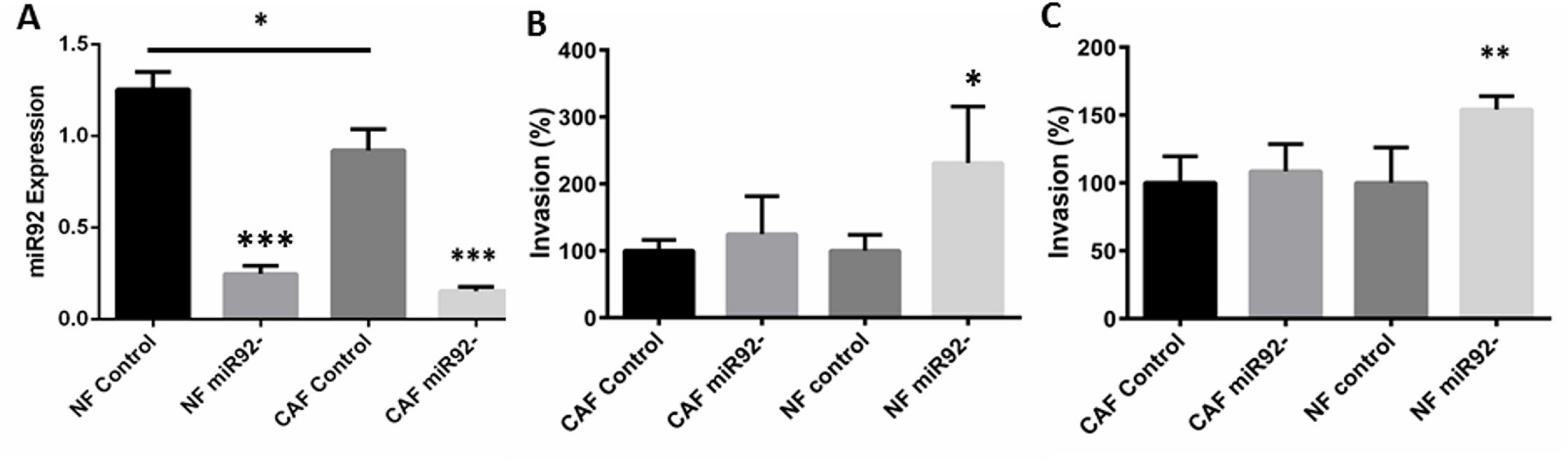
Down-regulation of miR92 expression in normal fibroblasts (NFs), but not cancer associated fibroblasts (CAFs), significantly enhances the invasive capacity of breast cancer epithelial cells. Matched NFs and CAFs were reverse transfected with either an inhibitor of miR92 or negative control (A) and a Matrigel™ invasion assay was used to assess effects on the behaviour of breast cancer epithelial cells 48 hours post-transfection. Down-regulation of miR92 significantly increased the invasion of MCF7 (B) and MDA-MB–231 (C) cells. Error bars are ± S.D. *denotes significance of p<0.05; **denotes significance of p<0.01; ***denotes significance of p<0.001.

We next hypothesised that levels of miR–92 in NFs or CAFs directly defines the behaviour of breast cancer epithelial cells. First, we selected a matched pair of primary NFs and CAFs with a high base line expression of miR–92 (p<0.05; Fig 5A) and reverse transfected these cells with an inhibitor for miR–92 or a non-targeting control to significantly reduce expression (p = 0.006 and p = 0.0057 in NFs and CAFs respectively; Fig 5A). We then used a Matrigel™ invasion assay to assess effects of transfected NFs or CAFs on the invasive capacity of MCF7 and MDA-MB–231 breast cancer epithelial cells growing on Matrigel™-coated membranes of Tranwell inserts placed in the dishes which contained the transfected fibroblasts. We found that down-regulation of miR–92 expression in NFs, but not CAFs, significantly increased the invasion of both MCF7 (Fig 5B) and MDA-MB–231 (Fig 5C) breast cancer epithelial cells (p = 0.011 and p = 0.0070, respectively). Representative images of the invasion assays for each cell line are shown in S1 Fig. Our data support a functional role for miR–92 in fibroblasts and show that low levels of miR–92 promote a more aggressive breast cancer phenotype in cell lines representing 2 different molecular subtypes of breast cancer suggesting that changes in expression of this molecule in NFs can directly impact upon the behaviour of breast cancer epithelial cells.

**Fig 6:**
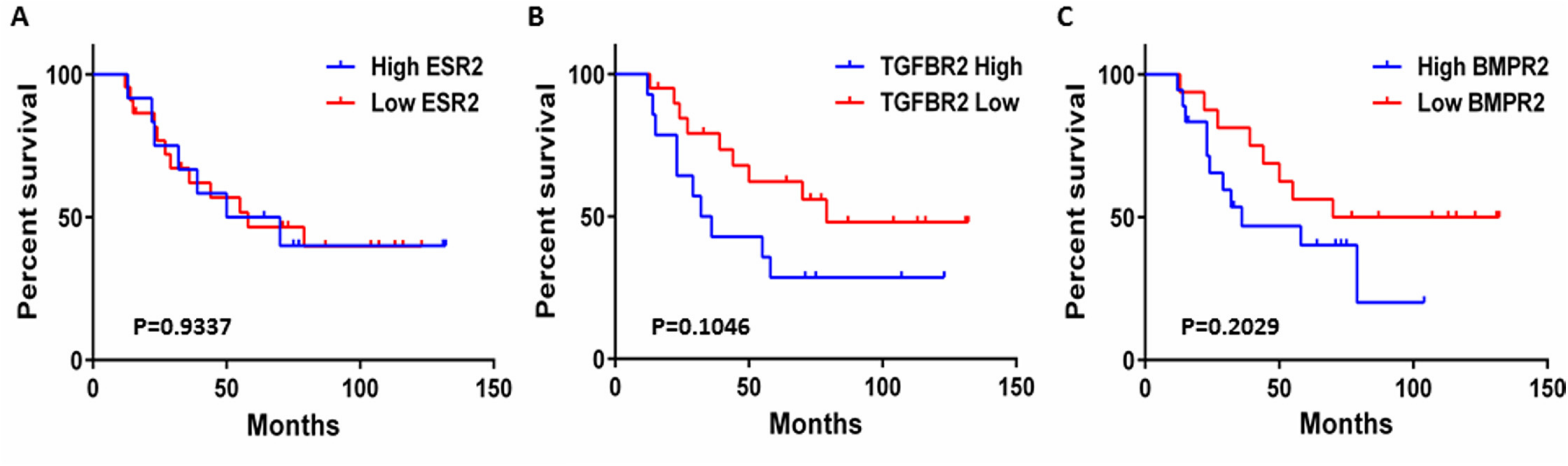
*In silico* analyses using the Oncomine platform showed that expression of the ESR2 gene in the breast tumour microenvironment does not correlate with patient outcome (p = 0.9337) (A). Expression of TGFBR2 (B) and BMPR2 (C) showed a trend towards significance (p = 0.1046 and p = 0.2029) suggesting that high expression levels of these genes are associated with lower rates of patient survival.

We then asked whether we could identify any potential targets through which miR–92 may be functioning in the breast tumour stroma. We identified several validated targets of miR–92 from the miRTarBase [30] and TarBase v6 [31] databases, including ERβ1 (S1 Table), confirming our previous work [12]. We performed further *in silico* analyses to correlate expression of several validated targets of miR–92 in the breast tumour stroma with patient survival using the Oncomine platform. We used data from Boersma et al. [27]since this was a relevant gene expression study identifying a stromal gene signature associated with patient outcome in inflammatory breast cancers (IBC) versus non-IBCs and we had acquired survival data directly from the corresponding author. For the purpose of our study, we removed the IBC cases (n = 13) and focused our mining of expression data for non-IBC patients (n = 34) to reduce potential confounding factors. We found that ESR2 (estrogen receptor β) expression within the breast cancer stroma was not significantly associated with patient outcome (p = 0.9337, Fig 6A) indicative that miR–92 may be functioning via the regulation of alternative targets. However the expression of two other miR–92 targets, TGFBR2 and BMPR2 showed a non-significant trend, potentially indicating that high expression levels of these genes may be associated with lower rates of patient survival (p = 0.1046 and p = 0.2029, respectively, Fig 6B and 6C).

### Expression of miR–92 Is Not Regulated by Epigenetic Mechanisms in Breast Cancer Epithelial Cells or Fibroblasts

Having shown significant differences in the absolute expression levels of miR–92 in both the breast epithelium and stromal compartments between individual patients, we performed bisulphite sequencing in a panel of breast cancer cell lines and primary fibroblasts to determine whether expression of this miRNA may be regulated epigenetically. We failed to detect methylation of CpG islands located within the promoter region of the miR-17-92 cluster (S2 Fig). We therefore concluded that alternative mechanisms of regulation were responsible for down-regulation of miR–92 expression in breast cancer epithelial cells and fibroblasts during cancer progression.

## Discussion

The oncogenic role for miRs encoded by the miR-17-92 cluster has been well documented in both haematological malignancies and solid tumours. Evidence has shown these miRs to be involved in the regulation of cell proliferation, apoptosis and angiogenesis [7, 8] by repressing transcripts including p63 [9], Bim [10] and components of the TGF-β pathway [11]. O’Donnell et al. have also shown that this cluster is a direct transcriptional target of c-myc, a transcription factor that is frequently hyperactive in human cancers [32]. Other lines of evidence using expression profiling techniques have revealed widespread overexpression of these miRs in various tumours including those of the breast. In accordance, our previous data has shown that ERβ1, a potential tumour suppressor gene in breast cancer, is a direct target of miR–92 and we found their expression levels to be inversely correlated in unselected non-microdissected breast tissues, providing a possible mechanism for the down-regulation of ERβ1 during cancer progression [12].

Importantly, in this study we found through *in silico* analysis [26] that luminal A breast cancer patients with high expression levels of miR–92 have a higher DFS compared to those with low expression levels. This has also been confirmed by Nilsson and colleagues [13, 33]. In accordance, LMD of specific epithelial areas from the same tissue showed that miR–92 expression levels do not increase during breast cancer progression, as expected for an onco-mir and negative regulator of ERβ1, but rather decrease in expression. This trend was seen in all but 1 case analysed. Interestingly, the pathological characteristics of this case differed from the others in that this was the only ER-negative case included in the cohort (Table 1). This may suggest that this phenomenon is dependent upon ER status, however requires analysis of a larger number of ER-negative samples to test this hypothesis. Given the well-recognised labour intensive nature of LMD [33], the analysis of a larger cohort of patient samples was not possible. Previously we have shown miR–92 levels were up-regulated in breast tumours compared with matched adjacent normal tissues [12]. However, researchers are now beginning to appreciate that there are often alterations in gene expression in adjacent normal breast tissue [34, 35]. Furthermore, LMD eliminates the theoretical possibility of signals, which may be subtle, being masked by influences from other cell types within tissues. Although not necessarily directly comparable to levels in tissue, recent work has examined the impact of circulating miR–92 in breast cancer; one showed this was reduced significantly in tissue and serum samples from breast cancer patients compared to those of healthy controls [36], while another showed miR–92 was elevated in the serum of breast cancer patients [37].

Despite the wealth of evidence for an oncomeric role for miR–92, there exists some contradictory evidence indicating that loss of function might be important in some cancer cells. Loss-of-heterozygosity (LOH) at the human genomic locus encoding the miR-17-92 cluster, 13q31.3, has been observed in several tumour types and a recent genome-wide analysis of copy number alterations in cancer revealed that this locus was deleted in 16.5% of ovarian cancers, 21.9% of breast cancers and 20% of melanomas [38]. Likewise, it has been shown that miR–92 can act as a tumour suppressor in breast tumours by inhibiting expression of AIB1 and/ or cyclin D1 [39, 40]. Nilsson et al. also found that expression of miR–92 was inversely associated with tumour grade in 144 cases of primary breast cancer and added independent prognostic information; patients with high levels of miR–92 had a better clinical outcome than patients with low levels [13]. Supporting data from this study also showed that down-regulation of miR–92 expression enhanced the migration of breast epithelial cells *in vitro* [13].

Interestingly, a shift in the localisation of ERβ1 staining from nuclear to cytoplasmic expression was observed by immunohistochemistry in breast epithelial cells. We were unable to confirm the expression levels and subcellular location of ERβ1 in our patient samples by western blotting. Although it is possible to assess subcellular location using western blotting, large quantities of protein are required and limited material was available by LMD. This material was also formalin-fixed; reliable quantitative protein analysis from formalin-fixed tissues can be problematic. Previous studies have shown that the localisation of ERβ isoforms can significantly influence prognosis in patients with breast [24] and ovarian [41] cancers. Our group examined cytoplasmic and nuclear expression of ERβ1, ERβ2 and ERβ5 in tissue microarrays comprising 842 cases of primary breast cancer and showed that patients with cytoplasmic ERβ2 expression had a significantly worse outcome compared to patients with nuclear staining [24]. A recent report by Ciucci et al. described an association between nuclear ERβ1 staining and low tumour grade in 95 patients with ovarian cancer suggesting the subcellular location of ERβ isoforms may influence their functional role within breast cancer cells [41]. It is possible that miR–92 may influence the subcellular expression of its targets via direct or indirect mechanisms. Chen and colleagues used microarray analyses to demonstrate that the nuclear-cytoplasmic ratio of a large panel of miRs varied considerably between cell lines representing normal breast (MCF-10A), non-invasive (MCF7) and invasive breast tissue (MDA-MB–231) [42]. In some cases the same miR presented similar overall expression levels in the 3 breast cell lines but showed distinct differences in their subcellular expression levels. This led the authors to speculate that de-regulated subcellular expression of the miRs themselves may be correlated with breast cancer progression, which may result in the abnormal subcellular expression of target mRNAs or proteins [42]. It is also possible that miR–92 may be indirectly influencing the subcellular localisation of targets by regulating the expression of transport and/or shuttling proteins.

There is growing recognition that the tumour stroma can impact upon tumour cell behaviour. Studies have also suggested that altered miR expression in CAFs might be a key regulator of breast tumour behaviour [1, 22]. Here, the pattern of miR–92 expression between matched CAFs and NFs seemed to fall into 2 groups; those with high miR–92 expression and those with low expression in NFs. Interestingly, elevated expression of miR–92 was observed in micro-dissected CAFs matched with NFs from the low-expressing group from patient samples. This trend was less apparent with primary NFs and CAFs however these cultures suffer from the drawback of not being exposed to the influences from other cell types present within the tumour microenvironment which may affect their behaviour and/ or expression profiles. This observation prompted us to ask whether fibroblasts with high or low levels of miR–92 expression could define the behaviour of breast cancer epithelial cells. We selected a matched case of NFs and CAFs with high expression of miR–92 so we could effectively inhibit expression to assess its effects. It is noteworthy that we did attempt the reverse experimentation via over-expression of miR–92 in a matched case of NFs and CAFs with low miR–92 expression, however, in accordance with previous observations, found this had an extremely toxic effect on these primary cells [1]. Our *in vitro* data confirmed that miR–92 can play a functional role in the tumour stroma and showed that fibroblasts with low miR–92 expression enhanced the invasion of breast cancer epithelial cells representing two different molecular subtypes of breast cancer. This effect was only seen for NFs and was not observed for CAFs. Analysis of baseline miR–92 expression in these cells revealed that NFs had a significantly higher level of miR–92 expression prior to inhibition, thus providing a possible explanation for the difference in outcome between NFs and CAFs. Nevertheless, this demonstrates that loss of miR–92 expression may have a role in regulating adjacent epithelial cell phenotype, acting via the stroma. In accordance, Nilsson et al. did not find a significant correlation between miR–92 expression and SMA-positivity in fibroblasts [13]. However, they found a strong inverse relationship with macrophage content, potentially indicating a role for miR–92 in the interaction between epithelial cells and immune cells in the tumour stroma compartment [13].

*In silico* data mining identified potential targets of miR–92 through which this miR may be mediating its effects in the breast stroma. We have previously shown ERβ1 is a target for miR–92 [12], however we found that expression of *ESR2* within the breast tumour stroma was not significantly associated with patient outcome. This was in accordance with results from our study that failed to show an inverse relationship between the expression of miR–92 and ERβ1 in the breast epithelium. However, it is worth highlighting that of the 5 different isoforms encode by *ESR2* only ERβ1 is a direct miR–92 target. It is therefore possible that this relationship may still exist in the breast stroma but is being masked by expression of more abundant isoforms within these cells. Indeed our previous work has shown expression of ERβ isoforms in fibroblasts was ranked: ERβ5 > ERβ2 > ERβ1 [24, 43]. Nevertheless, in the context of this study, these data are indicative that miR–92 may be functioning via the regulation of targets other than ERβ1. Interestingly, the expression of TGFBR2 and BMPR2 showed a trend towards significance and suggests that high expression levels of these genes are associated with lower rates of patient survival. We recognise the limitations of this observation and mining of a larger dataset may allow this trend to reach significance; following removal of the inflammatory breast cancer cases, it was possible to mine data from just 34 patients with invasive ductal breast cancer. Nevertheless, these observations are in accordance with our data, which suggest low levels of miR–92 are associated with enhanced invasion and a more aggressive tumour phenotype. Low levels of miR–92 would lead to an increase in the expression of TGFBR2 and BMPR2 and lead to a poorer patient prognosis. The TGFBR2 gene encodes for a member of the Ser/Thr protein kinase family and has a tumour suppressor or promoter role depending on cellular context [44]. Busch et al. analysed the expression of TGFBR2 in CAFs in a cohort of 252 invasive breast cancers and found that CAF-specific TGFBR2 expression correlated with improved recurrence-free survival. Experimentally, they also showed that knock-down of TGFBR2 in CAFs resulted in increased cell growth, proliferation and clonogenic survival of breast cancer cells and suggest that regulation of tumour-stromal cross-talk through fibroblastic TGF-β pathway may depend on fibroblast phenotype. The BMPR2 gene also encodes for a Ser/Thr receptor kinase binding bone morphogenetic proteins as well as members of the TGF-β superfamily of ligands. The role of BMPR2 remains unclear and reports suggest that this protein can also have tumour-suppressing and -promoting roles within different cells. Inhibition of BMPR2 has been shown to inhibit growth and viability of breast cancer cells [45]. In contrast, Owens et al. found that BMPR2 had a tumour-suppressive function in mammary epithelia and microenvironment and suggest that disruption can accelerate mammary carcinoma metastases [46].

Finally, it is known that epigenetic mechanisms including DNA methylation and histone modification contribute to expression of some miRNAs, including those of the miR-17-92 cluster [47–51]. Epigenetic regulation of miRNA expression has been described in colorectal, breast and lung cancers. Here, we hypothesised that epigenetic mechanisms may contribute to the regulation of miR–92 expression, however we did not detect methylation of CpG islands within the promoter region located upstream of the miR-17-92 cluster in a range of breast cancer cell lines or in primary fibroblasts, suggesting other mechanisms are responsible for loss of miR–92 during breast cancer progression.

## Conclusions

We have shown loss of expression of miR–92 in breast epithelial cells during breast cancer progression with a corresponding shift in expression of one of its targets, ERβ1 from a nuclear to a cytoplasmic location. This may represent a potential novel mechanism by which miRs can influence the function of their targets. However *in silico* analysis suggests that ERβ1 may not be the most important miR92 target in breast cancer. Finally, while miR–92 expression levels remained unchanged in stromal fibroblasts, our data support a functional role for this molecule in fibroblasts and show that down-regulation of miR–92 expression in NFs can influence the invasive capacity of breast cancer epithelial cells.

## Supporting Information

S1 Fig Invasive capacity of MCF–7 (a-d) and MDA-MB–231 (e-h) cells in response to NFs (a, b, e, f) or CAFs (c, d, g, h) using a Transwell assay. NFs in which miR92 was silenced (b, f) enhanced invasion in both cell lines compared to non-silenced controls (a, e), while no effect was seen in either cell line when miR92 was silenced in CAFS (d, h), compared to respective controls (c, g). Under control conditions MDA-MB–231 (e, g) cells showed greater invasive capacity than MCF–7 (a, c), as predicted for this cell line. At the end of the assay, cell invasion through the Transwell™ membranes was visualised using crystal violet staining.
(PDF)

S2 Fig Pyrosequencing revealed no methylation of CpG islands within the promoter region of the miR-17-92 cluster in a panel of breast cell lines or in breast primary normal and cancer-associated fibroblasts (PDF)

S1 Table Identification of the top 10 validated targets of miR–92 identified from miRTarBase [30] and TarBase v6 [31] databases. (PDF)

## Abbreviations

BMPR2: bone morphogenetic protein receptor II
CAF: cancer-associated fibroblast
DCIS: ductal carcinoma in situ
DFS: disease free survival
DMEM: Dulbecco’s modified eagle medium
ER: estrogen receptor
FBS: foetal bovine serum
FFPE: formalin-fixed paraffin-embedded
HER: human epidermal growth factor receptor
IBC: inflammatory breast cancer
LMD: laser microdissection
LOH: loss-of-heterozygosity
miR: microRNA
NF: normal fibroblast
PTEN: phosphatase and tensin homolog
qRT-PCR: quantitative polymerase chain reaction
RPMI: Roswell Park Memorial Institute
SDF–1: stromal cell-derived factor–1
SMA: smooth muscle actin
TGF: transforming growth factor
TGFBR2: transforming growth factor beta receptor II
UTR: untranslated region
