# Supplementary material for: Down-Regulation of miR-92 in Breast Epithelial Cells and in Normal but Not Tumour Fibroblasts Contributes to Breast Carcinogenesis
Source: PLoS One. 2015 Oct 5;10(10):e0139698. doi: 10.1371/journal.pone.0139698 (PMC4593575; doi:10.1371/journal.pone.0139698)
Supplement: S1 Fig — NFs in which miR92 was silenced (b, f) enhanced invasion in both cell lines compared to non-silenced controls (a, e), while no effect was seen in either cell line when miR92 was silenced in CAFS (d, h), compared to respective controls (c, g). Under control conditions MDA-MB–231 (e, g) cells showed greater invasive capacity than MCF–7 (a, c), as predicted for this cell line. At the end of the assay, cell invasion through the Transwell™ membranes was visualised using crystal violet staining. (PDF) [file pone.0139698.s001.pdf]

S1 Fig

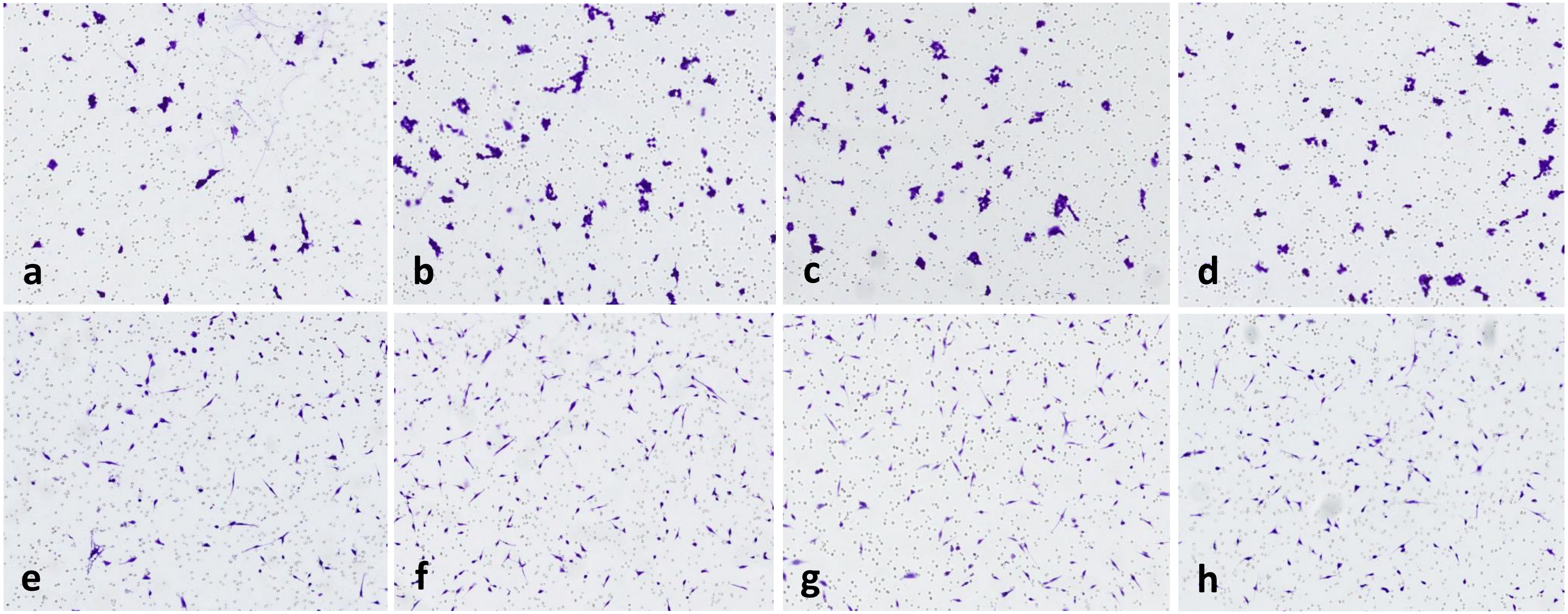

Invasive capacity of MCF-7 (a-d) and MDA-MB-231 (e-h) cells in response to NFs (a, b, e, f) or CAFs (c, d, g, h) using a Transwell assay. NFs in which miR92 was silenced (b, f) enhanced invasion in both cell lines compared to non-silenced controls (a, e), while no effect was seen in either cell line when miR92 was silenced in CAFS (d, h), compared to respective controls (c, g). Under control conditions, MDA-MB-231 (e, g) cells showed greater invasive capacity than MCF-7 (a, c), as predicted for this cell line. At the end of the assay, cell invasion through the Transwell™ was visualised using crystal violet staining.
