# Supplementary material for: Down-Regulation of miR-92 in Breast Epithelial Cells and in Normal but Not Tumour Fibroblasts Contributes to Breast Carcinogenesis
Source: PLoS One. 2015 Oct 5;10(10):e0139698. doi: 10.1371/journal.pone.0139698 (PMC4593575; doi:10.1371/journal.pone.0139698)

S2 Fig

Pyrosequencing revealed no methylation of CpG islands within the promoter region of the miR-17-92 cluster in a panel of breast cancer cell lines and primary normal- and cancer-associated fibroblasts

Assay Name: Mir17 CpG assay 1739-2047
Sample ID: Primary normal fibroblasts from patient 1

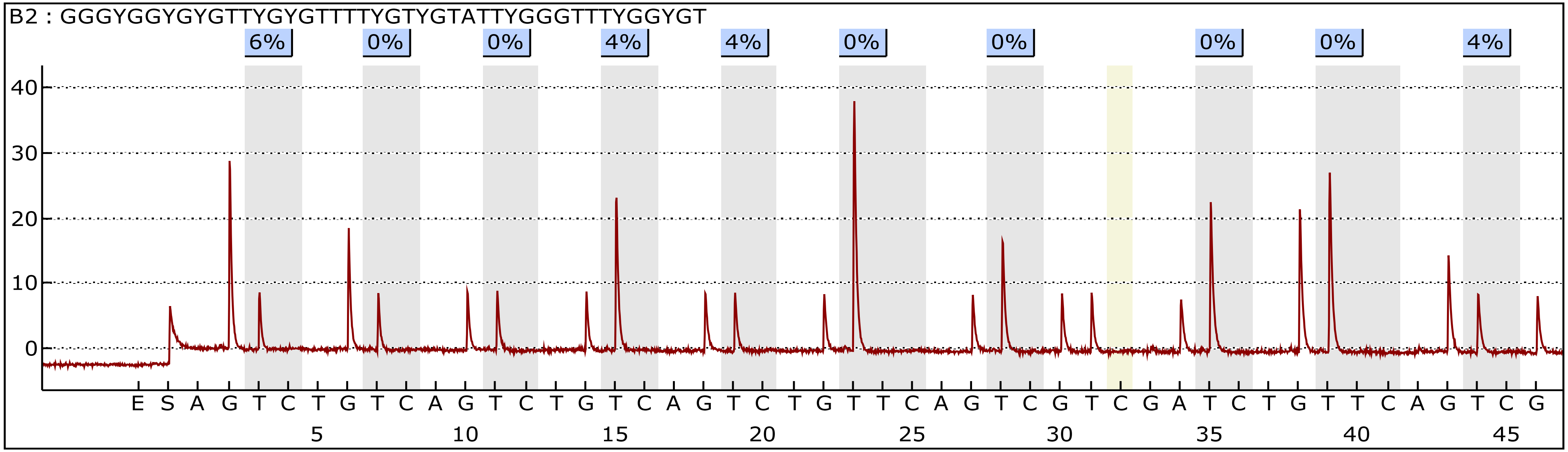

Assay Name: Mir17 CpG assay 1739-2047
Sample ID: Primary cancer-associated fibroblasts from patient 1

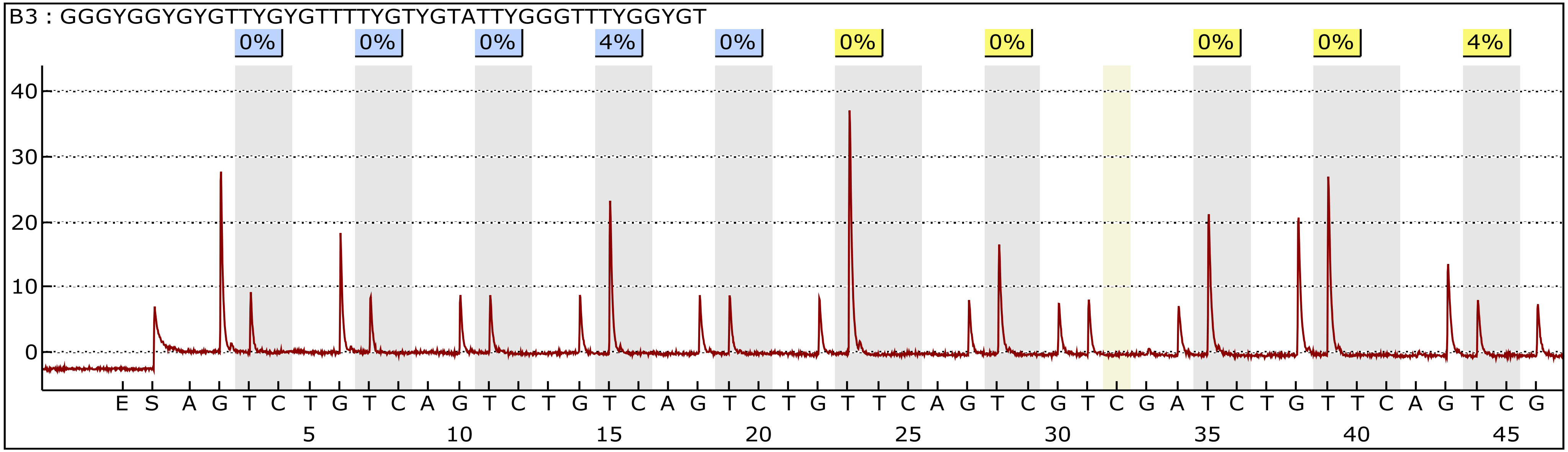

Assay Name: Mir17 CpG assay 1739-2047
Sample ID: Primary normal fibroblasts from patient 2

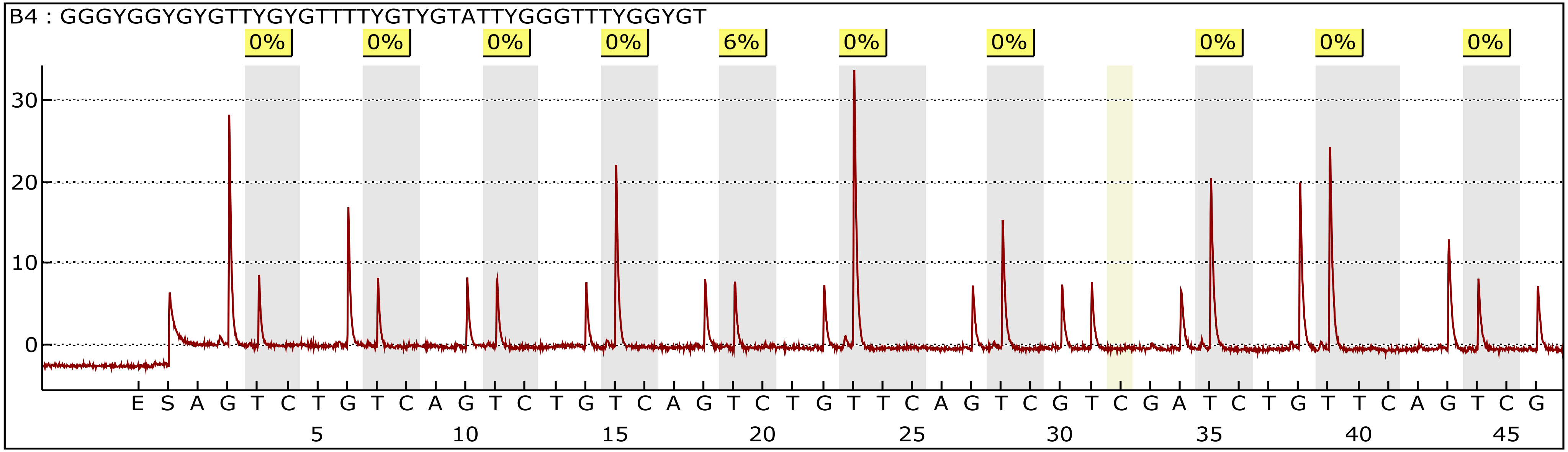

Assay Name: Mir17 CpG assay 1739-2047
Sample ID: Primary cancer-associated fibroblasts from patient 2

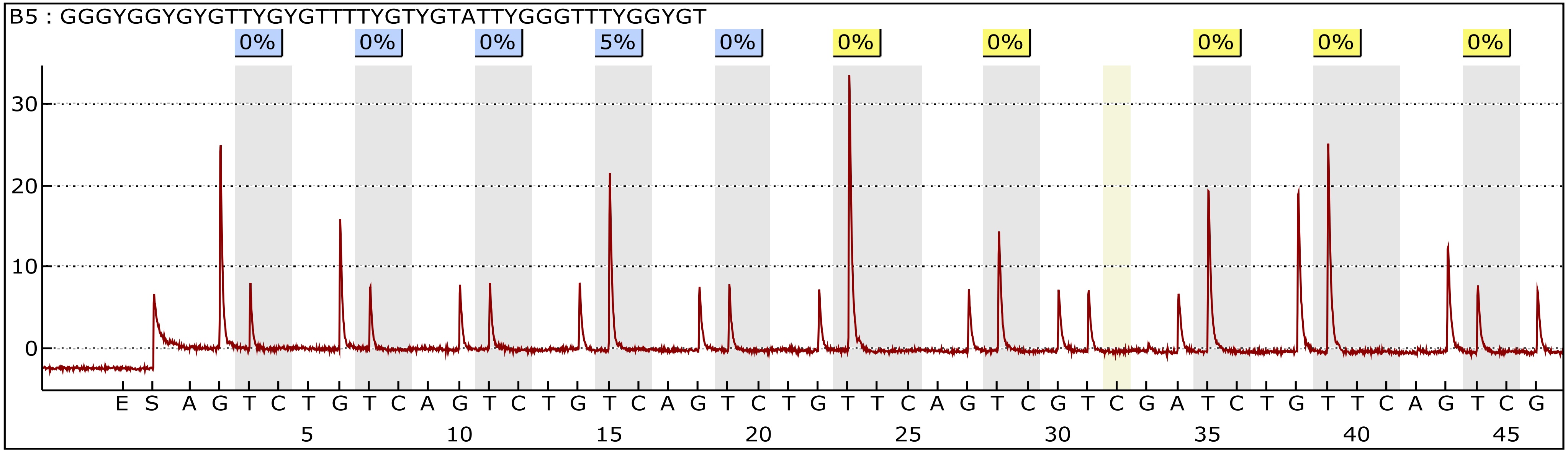

Assay Name: Mir17 CpG assay 1739-2047
Sample ID: HB2 cell line

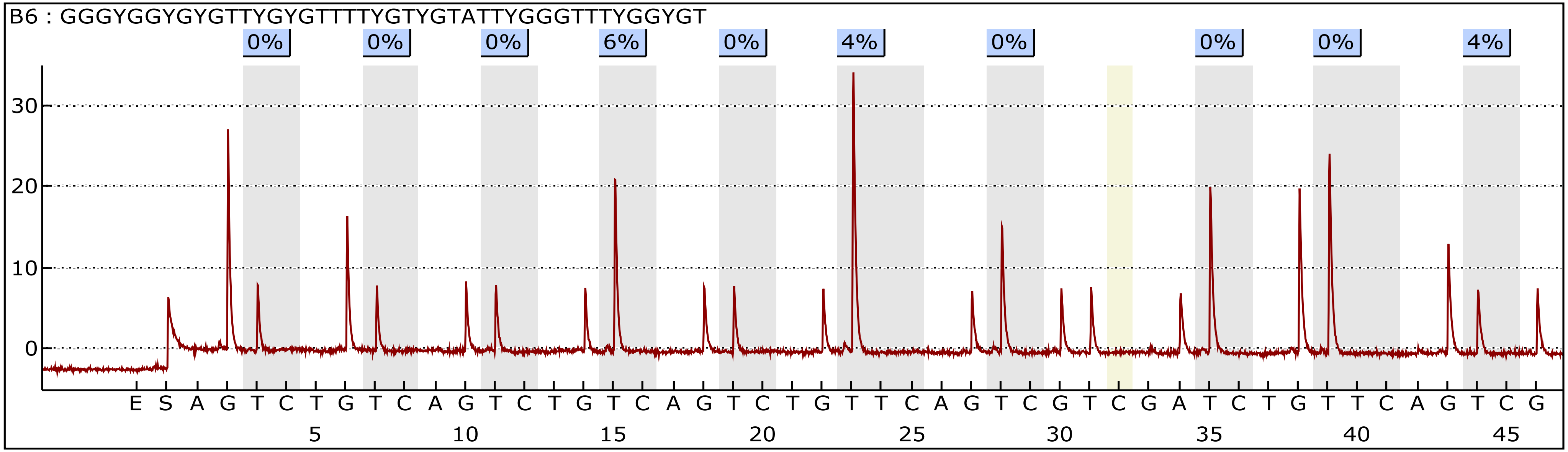

Assay Name: Mir17 CpG assay 1739-2047
Sample ID: BT474 cell line

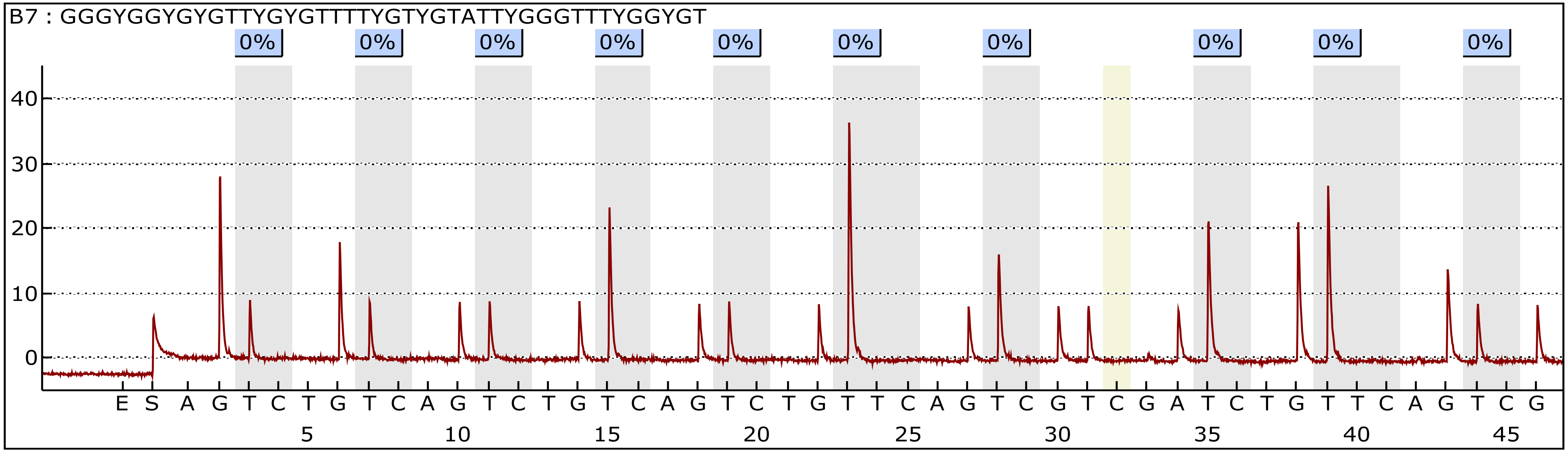

Assay Name: Mir17 CpG assay 1739-2047
Sample ID: T47D cell line

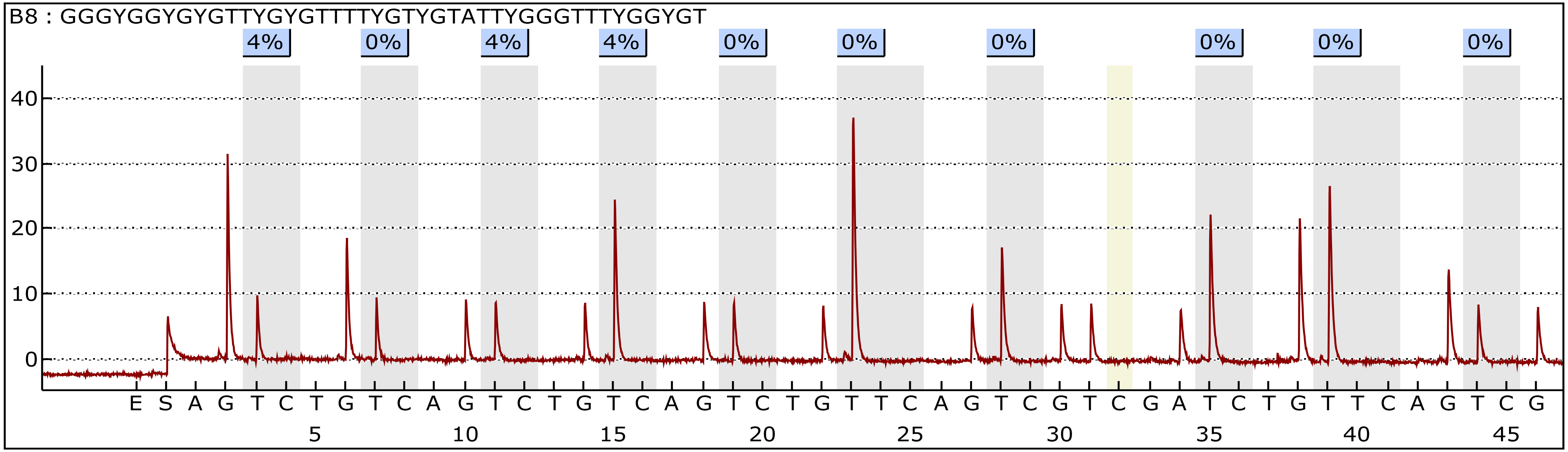

Assay Name: Mir17 CpG assay 1739-2047
Sample ID: MDAMB453 cell line

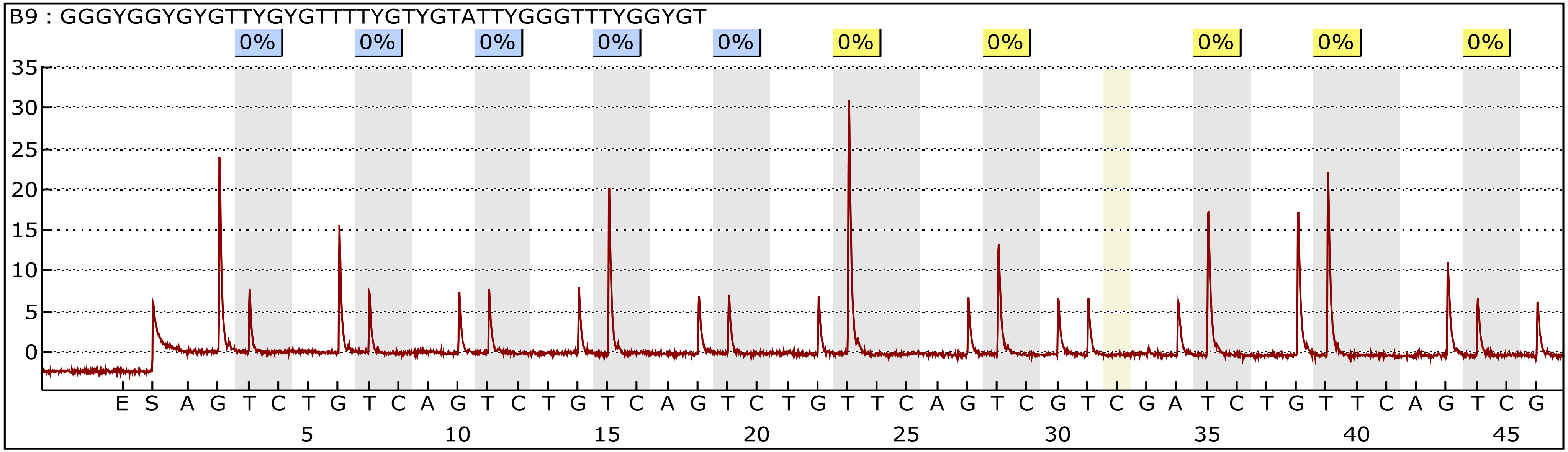

Assay Name: Mir17 CpG assay 1739-2047
Sample ID: BT20 cell line

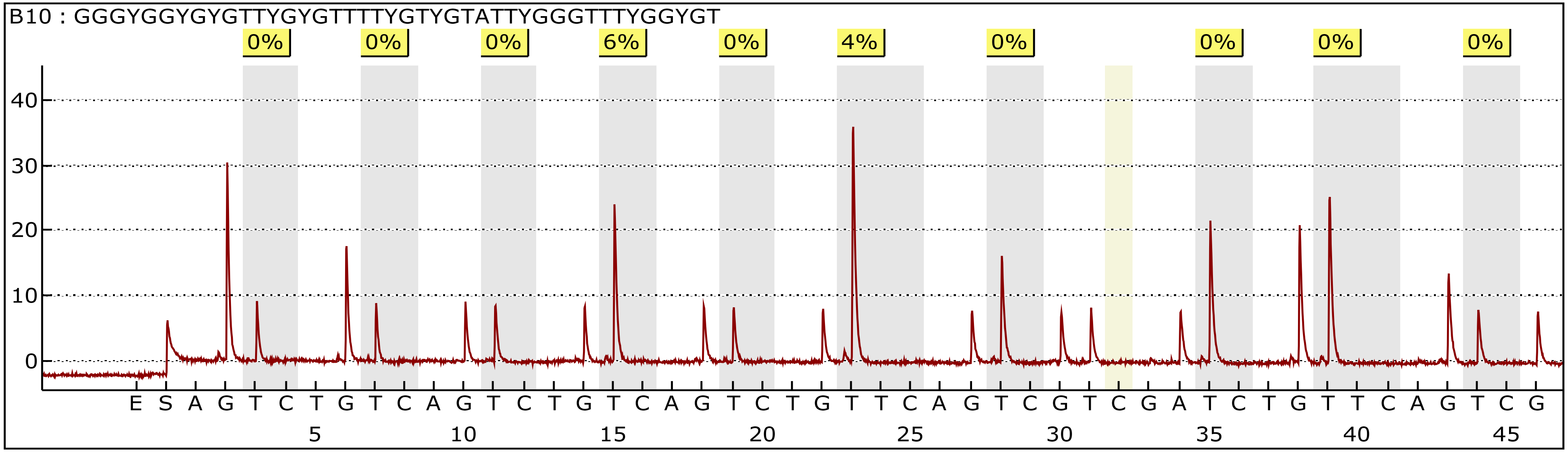

Assay Name: Mir17 CpG assay 1739-2047
Sample ID: MDAMB468 cell line

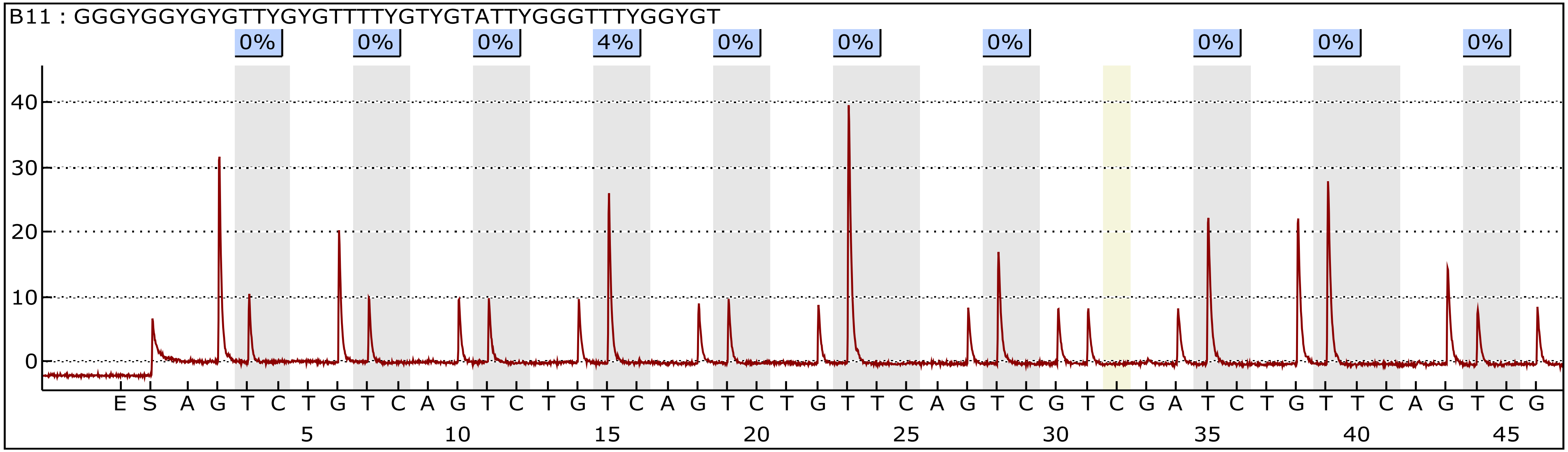

Assay Name: Mir17 CpG assay 1739-2047
Sample ID: MCF7 cell line

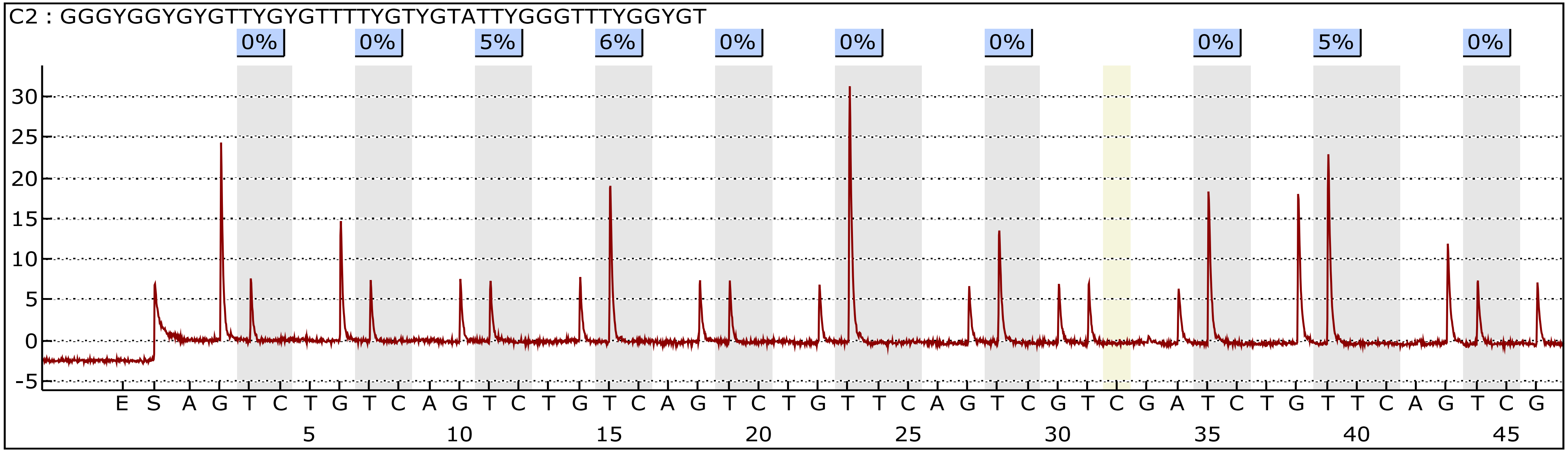

Assay Name: Mir17 CpG assay 1739-2047
Sample ID: C0971 gDNA control

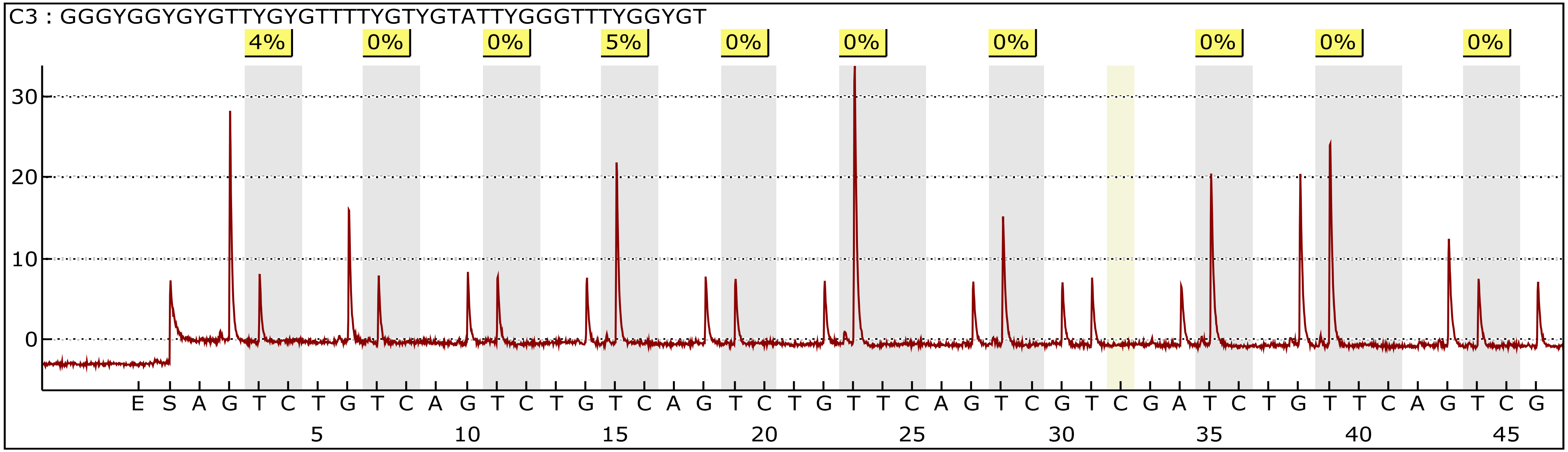

Assay Name: Mir17 CpG assay 1739-2047
Sample ID: QA2 gDNA control

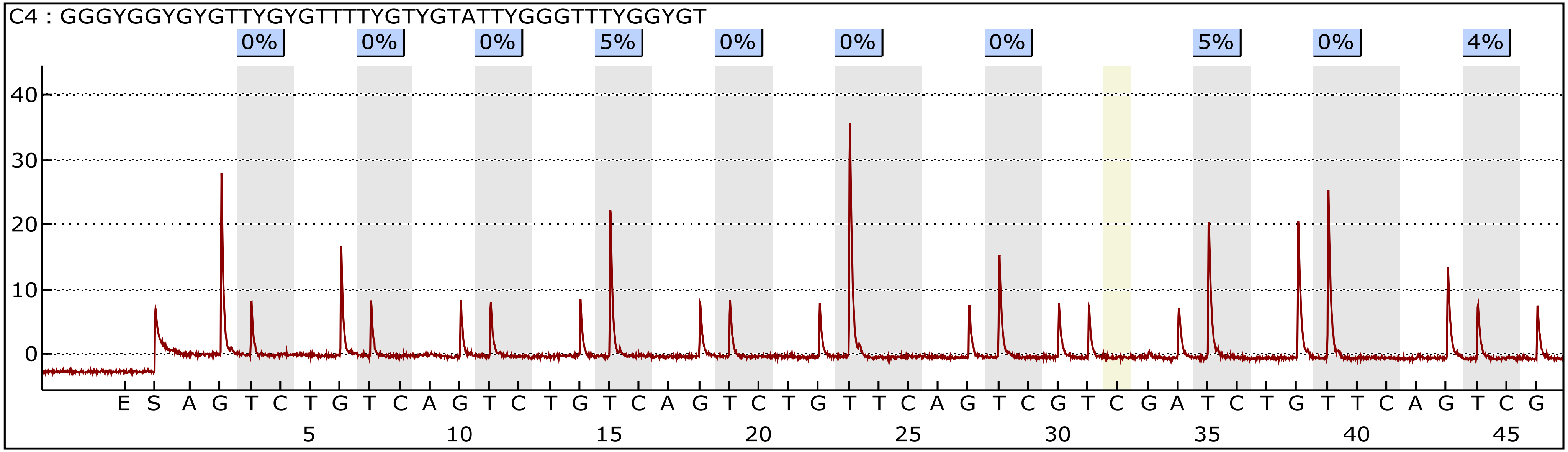

Assay Name: Mir17 CpG assay 1739-2047
Sample ID: no template control

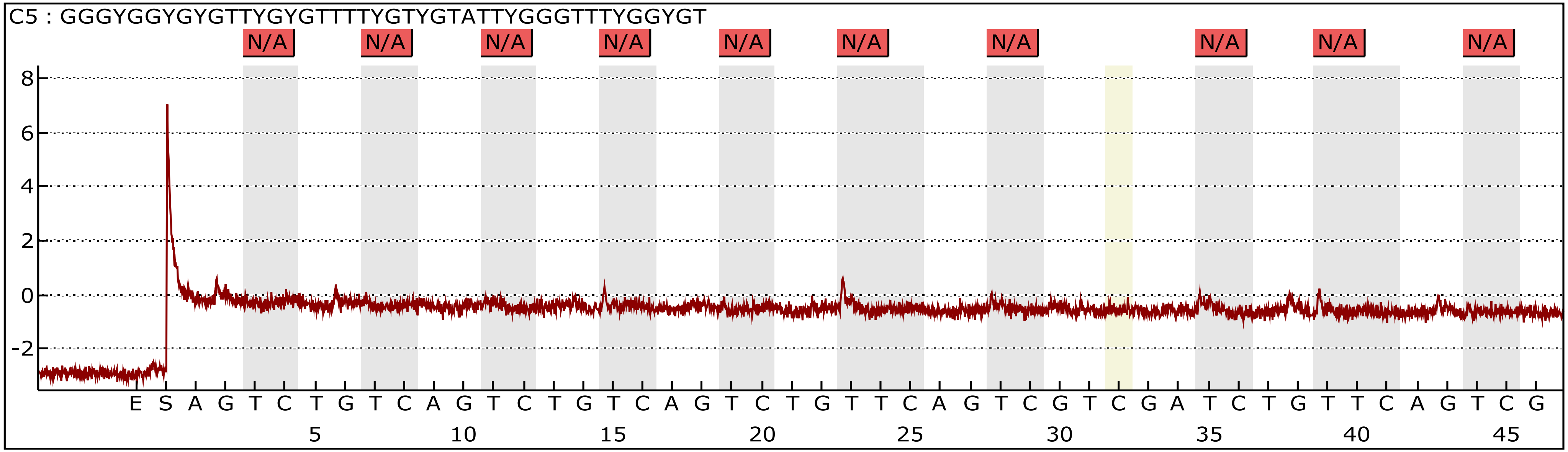

Supplement: S2 Fig — (PDF) [file pone.0139698.s002.pdf]
