# Supplementary material for: Down-Regulation of miR-92 in Breast Epithelial Cells and in Normal but Not Tumour Fibroblasts Contributes to Breast Carcinogenesis
Source: PLoS One. 2015 Oct 5;10(10):e0139698. doi: 10.1371/journal.pone.0139698 (PMC4593575; doi:10.1371/journal.pone.0139698)
Supplement: S1 Table — (PDF) [file pone.0139698.s003.pdf]

Supplementary Table 1

Target Gene	Function
ITGA5	Receptor for fibronectin and fibrinogen
ARID4B	Transcriptional repressor
HIPK3	Ser/Thr protein kinase involved in transcription regulation
MYLIP	E3 ubiquitin-protein ligase
TP63	A member of the p53 family of transcription factors
KAT2B	A histone acetyltransferase (HAT) to promote transcriptional activation
ESR2	Estrogen receptor family member/ nuclear receptor transcription factor superfamily member
TGFBR2	Ser/Thr protein kinase/ TGF-β receptor subfamily member
BMPR2	Ser/Thr protein kinase/ bone morphogenetic protein (BMP) receptor family member
CPEB2	Similar to cytoplasmic polyadenylation element binding protein (CPEB), an mRNA-binding protein that regulates cytoplasmic polyadenylation of mRNA

Top 10 validated targets of miR-92 identified from miRTarBase and TarBase v6 databases
